# Synergistic activity of *Enterococcus Faeciu*m-induced ferroptosis via expansion of IFN-γ^+^CD8^+^ T cell population in advanced hepatocellular carcinoma treated with sorafenib

**DOI:** 10.1080/19490976.2024.2410474

**Published:** 2024-10-01

**Authors:** Haitao Yu, Ganglian Lin, Junyan Jiang, Jiangqiao Yao, Zhenyan Pan, Haonan Xie, Zhiyuan Bo, Qikuan He, Jinhuan Yang, Ziyan Chen, Jiacheng Li, Yi Wang, Zhengping Yu, Yehuda G. Assaraf, Gang Chen

**Affiliations:** a Department of Hepatobiliary Surgery, The First Affiliated Hospital of Wenzhou Medical University, Wenzhou, Zhejiang, China; b Key Laboratory of Diagnosis and Treatment of Severe Hepato-Pancreatic Diseases of Zhejiang Province, The First Affiliated Hospital of Wenzhou Medical University, Wenzhou, Zhejiang, China; c Zhejiang-Germany Interdisciplinary Joint Laboratory of Hepatobiliary-Pancreatic Tumor and Bioengineering, The First Affiliated Hospital of Wenzhou Medical University, Wenzhou, Zhejiang, China; d Alberta Institute, Wenzhou Medical University, Wenzhou, Zhejiang, China; e Department of Epidemiology and Biostatistics, School of Public Health and Management, Wenzhou Medical University, Wenzhou, Zhejiang, China; f The Fred Wyszkowski Cancer Research Laboratory, Department of Biology, Technion-Israel Institute of Technology, Haifa, Israel

**Keywords:** Hepatocellular carcinoma, sorafenib, gut microbiota, IFN-γ^+^CD8^+^ T cell, ferroptosis

## Abstract

The gut microbiota plays an important role in the development and treatment of hepatocellular carcinoma (HCC). However, the implication of specific gut microbiota in targeted sorafenib therapy for advanced HCC and the microbiota mode of action, remain to be elucidated. Here, we confirmed that four bacterial genera, *Lachnoclostridium*, *Lachnospira*, *Enterobacter* and *Enterococcus*, are associated with the therapeutic efficacy of Sorafenib, and that *Enterobacter faecium* (Efm) plays a crucial role in modulating the sorafenib activity. The effective colonization by Emf induced the IL-12 and IFN-γ production and an increased proportion of IFN-γ^+^CD8^+^ T cells in the tumor microenvironment. Finally, exopolysaccharides (EPS) from Efm were the primary inducer to prompt IFN-γ^+^CD8^+^ T cells to secrete IFN-γ, which together with sorafenib instigated ferroptosis in HCC cells. Collectively, these results indicate that Efm is a promising probiotics that enhances the efficacy of sorafenib treatment in advanced HCC.

## Introduction

Hepatocellular carcinoma (HCC) is the predominant form of primary liver cancer ranking sixth among the most frequently diagnosed cancers and fourth among the leading causes of cancer-related mortality worldwide. Furthermore, its incidence has increased globally over the past decade.^1^ HCC is a highly malignant tumor with a poor prognosis with radical resection being an effective treatment for it. Nonetheless, the majority of patients are diagnosed at an advanced stage,^2^ with only 9–27% of patients being suitable for surgical resection upon initial diagnosis.^3^ Extremely limited treatment options are available for patients with advanced HCC who cannot undergo surgical treatment; hence, the only effective treatment remains systemic chemotherapy.^4^ However, conventional chemotherapies, such as doxorubicin and 5-fluorouracil, exhibit poor therapeutic efficacy and serious side effects.^5^ Therefore, molecular-targeted agents (MTAs) are the primary treatment options for patients with unresectable HCC. Sorafenib, as the first-line multikinase inhibitor for the treatment of HCC, blocks tumor angiogenesis via vascular endothelial growth factor receptors (VEGFRs), platelet-derived growth factor receptor-β (PDGFRβ), Kit receptor tyrosine kinase and FMS-like tyrosine kinase 3 (FLT-3)^6^ and induces ferroptosis by inhibiting the system Xc^−^ function.^7^ Unfortunately, sorafenib resistance frequently emerges in HCC and only approximately 33% of patients with advanced HCC respond to treatment,^8^ highlighting the urgent need to enhance the sensitivity of HCC to sorafenib, thereby prolonging patient survival.

The gut microbiota is widely recognized as an important factors regulating the host health and strongly affects the metabolic, endocrine, and immune systems. Recent research has indicated that the gut microbiota is associated with several diseases such as obesity,^9^ nonalcoholic fatty liver disease,^10^ diabetes,^11^ and malignant tumors.^12–14^ In addition, the properties of gut microbiota vary among different diseases. The mutually beneficial association between the host and gut microbiota is a crucial factor in preserving gut homeostasis, which can regulate host metabolism and immunity to improve host physiology and health.^15^ Notably, gut dysbiosis can facilitate the onset and progression of tumors and affect the efficacy of tumor therapy. Increasing evidence exists regarding the relationship between gut microbiota and anticancer drug activity with certain gut microbiota strains enhancing the anti-tumor efficacy of drugs and alleviating the adverse effects of chemotherapy.^16,17^ For instance, *Enterococcus hirae* translocates from the gut to secondary lymphoid organs and augments the CD8/Treg ratio within the tumor microenvironment (TME), whereas the colonization of *Barnesiella intestinihominis* in the colon promotes the infiltration of intratumoral γδT cells producing IFN-γ during cyclophosphamide chemotherapy.^18^ Furthermore, a high abundance of *Lactobacillus reuteri* in intestine alleviates the intestinal damage caused by cisplatin and maintains gut microbiota balance.^19^ Although numerous studies have demonstrated a correlation between gut microbiota constituents and HCC,^20,21^ there is a lack of published research regarding gut microbiota characteristics in patients with unresectable HCC undergoing sorafenib treatment. Furthermore, it has not been determined whether there is an association between the gut microbiota and response to sorafenib. Thus, in the current study, we examined the characteristics of the gut microbiota of patients with advanced HCC with diverse responses to sorafenib. Our results indicated a clear accumulation of *Enterococcus faecium* (Efm) in the intestines of patients who responded positively to sorafenib compared to those who showed no response to sorafenib. In addition, subsequent experiments revealed that the combination of Efm and sorafenib was capable of suppressing tumor cell proliferation and enhancing the frequency of intratumoral IFN-γ^+^CD8^+^ T cells. This tumor-suppressing effect was attributed to the exopolysaccharides from Efm, which increased the percentage of IFN-γ^+^CD8^+^ T cells and synergized with sorafenib to induce ferroptosis in HCC cells.

## Results

### Clinical data and gut microbiota of patients with different treatment responses

**Figure 1.**
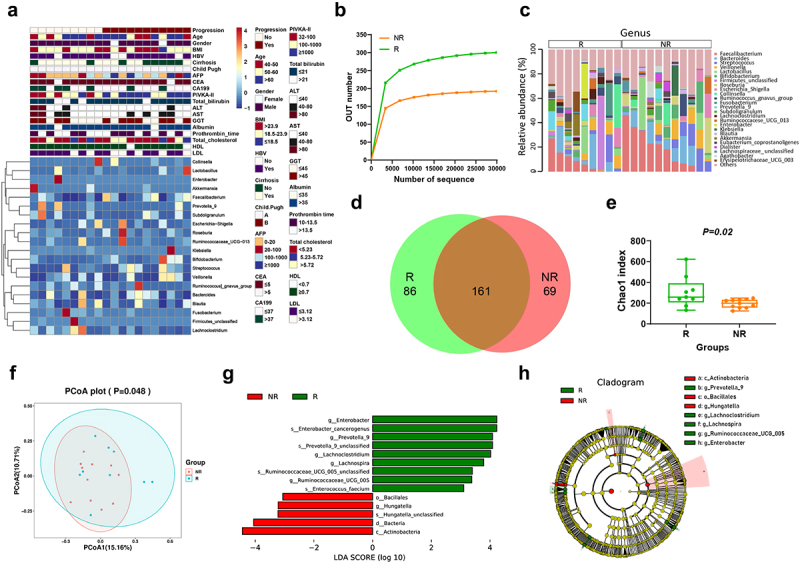
Clinicopathological data and gut microbiota characteristics of all patients. (a) Clinicopathological data and the top 20 gut microbiota at genus level of all 20 patients with advanced hepatocellular carcinoma. (b) rarefaction plots, operational taxonomic units at increasing sequencing depth in responders (R) group and non-responders (NR) group. (c) Compositions of bacterial community at the genus level between two groups. (d) A venn diagram illustrates that 316 of the total richness of 161 OTUs were shared among two groups. (e) α-diversity was measured by Chao1 index in R and NR group. (f) Principal coordinate analysis of gut microbiomes using the unifrac dissimilarity. (g) LDA scores computed for differentially abundant taxa (LDA>3) in the microbiomes of responders (green) and non-responders (red). (h) Taxonomic cladogram from LEfSe showing differences among taxa between two groups. Each dot represents a patient. Error bars represent the SEM. OTUs, operational taxonomic units; LDA, linear discriminant analysis; LEfSe, linear discriminant analysis effect size.

Twenty patients with advanced HCC were enrolled in this study; of which 9 patients responded to sorafenib, whereas 11 patients progressed after more than 3 months of sorafenib administration. All the patients lived in Wenzhou and practiced comparable dietary habits. The clinicopathological data including the age, gender, BMI, HBV, liver cirrhosis, Child Pugh, CEA, CA-199, PIVKA-II, total bilirubin, ALT, AST, γ-GT, albumin, prothrombin time, total cholesterol, HDL and LDL of the two groups (responders, R, *n* = 9 and non-responders, NR, *n* = 11) of patients were not significantly different. Serum AFP was significantly elevated in the non-responder group compared to those in the responder group (Figure 1a and Table S1). The 16S rRNA analysis was performed on the fecal microbiota of the two groups, and rarefaction analysis showed that the estimated richness of operational taxonomic units (OTUs) in the two groups was stable (Figure 1b). The composition of the bacterial community (top 25) at the genus level is depicted in Figure 1c. The Venn diagram illustrates that 161 bacteria at the genus level were shared between these two groups, whereas 89 out of 316 bacteria were solely present in the responders group (Figure 1d). The fecal microbial α-diversities analysis showed that the chao1 index was significantly increased in the responders group (Figure 1e), and β-diversities analysis illustrated that there were differences in the composition of gut microbiota between the two groups (Figure 1f). Linear discriminant effect size (LEFSe) analysis was used to explore differences in gut microbiota between the two groups. In terms of effect size, *Enterobacter (g)*, *Prevotella_9 (g)*, *Lachnoclostridium (g)*, *Lachnospira (g)*, *Ruminococcaceae_UCG_005 (g)*, *Enterobacter_cancerogenus (sp)*, *Enterococcus_faecium (sp)*, *Prevotella7_9_unclassified (sp)* and *Ruminococcaceae_UCG_005_unclassified (sp)* were significantly enriched in the responders group compared to the non-responders group (Figure 1g). The cladogram shows similar results (Figure 1h).

### Efm enhances the activity of sorafenib against HCC

**Figure 2.**
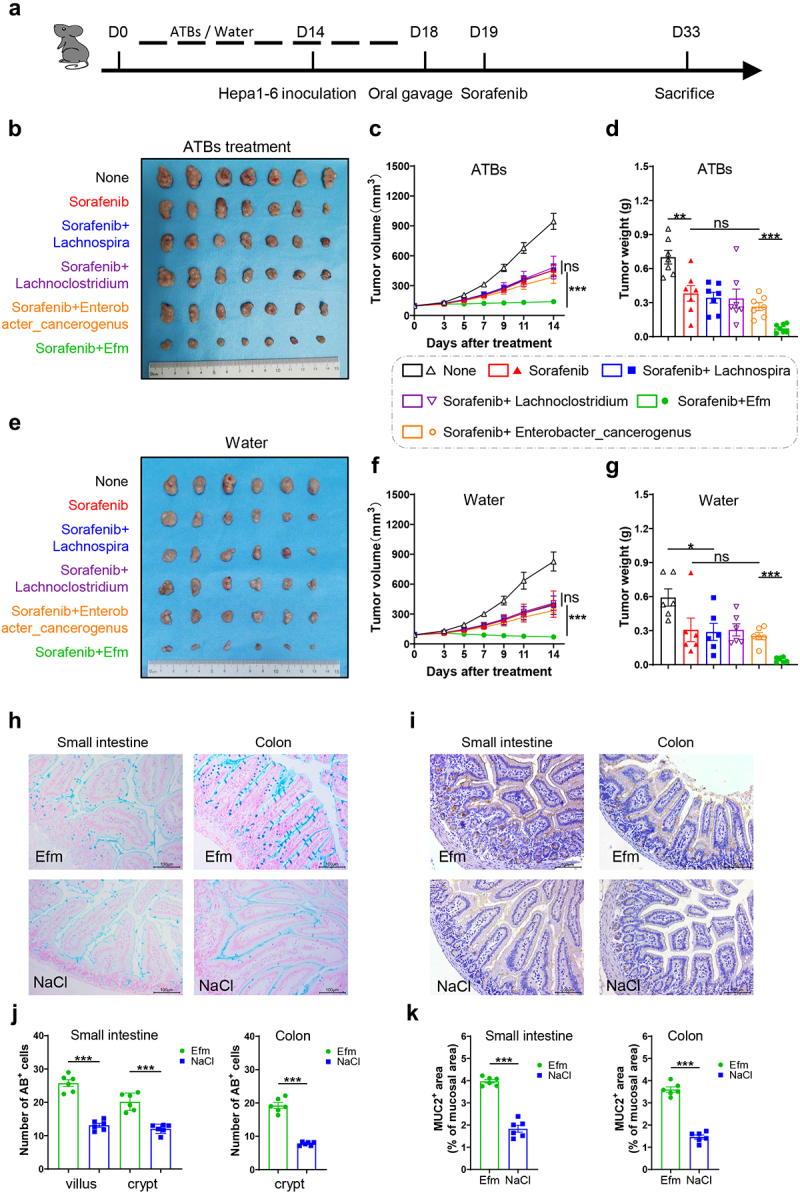
Efm enhanced the anti-tumor ability of sorafenib against hepatocellular carcinoma and protected the intestinal barrier function. (a) Schematic diagram of the experimental design of various bacterial groups combined with sorafenib on hepatocellular carcinoma with or without ATBs treatment. (b) Images of subcutaneous tumors from hcc-bearing mouse model with ATBs pretreatment under different treatments. (c-d) tumor volume and tumor mass in mouse model with ATBs pretreatment under different treatments. (e) Images of subcutaneous tumors from hcc-bearing mouse model without ATBs pretreatment under different treatments. (f,g) tumor volume and tumor mass in mouse model without ATBs pretreatment under different treatments. (h) The number of goblet cell in small intestine and colon were visualized using Alcian blue staining. (i) Muc-2 expression in small intestine and colon assessed by immunohistochemistry staining. (j) Oral gavage with efm promoted the recovery goblet cells in villi and crypt of the small intestine and crypt of the colon after ATBs treatment. (k) Oral gavage with efm increased muc-2 expression in the small intestine and colon. Each dot represents a mouse. Error bars represent the SEM. ns *p* > 0.05, *p < 0.05, **p < 0.01, ***p < 0.001.

Seven bacterial strains from four genera were used in the experiments to investigate which bacterium plays a key role in HCC treatment with sorafenib. First, we colonized the gut microbiota of hepa1-6 HCC-bearing mice that received broad-spectrum antibiotic administration for 14 days or only water using four genera of bacteria (Figure 2a): *Lachnospira multipara, Lachnospira eligens, Lachnoclostridium bouchesdurhonense, Lachnoclostridium massiliosenegalense, Lachnoclostridium touaregense, Enterococcus faecium* and *Enterobacter_cancerogenus*. Intestinal bacteria in mice decreased significantly on the third day of antibiotic treatment (Figure S1A and S1B). However, after 10 days of antibiotic treatment, the mice showed no further reduction in gut bacteria compared to that on the third day (Figure S1C). The gut bacteria of antibiotic-treated mice began to recover after 3 days (Figure S1D and S1E) of bacterial supplementation and normalized after 10 days (Figure S1F). Treatment with sorafenib followed by Efm effectively inhibited the tumor growth in mice following antibiotic administration (Figure 2b,c), and the tumor mass was significantly decreased -compared to that in the other groups (Figure 2d). Similar results were obtained when these bacteria were supplemented in mice with normal gut microbiota (Figure 2e) as the volume (Figure 2f) and mass (Figure 2g) of subcutaneous tumors in mice supplemented with Efm were smaller than those in the other groups. Moreover, on days 7 and 14, no difference was observed in the growth of subcutaneous tumors between mice treated with antibiotics and those treated with normal gut bacteria (Figure S1G and S1H). Additionally, the administration of Efm resulted in increased proliferation of goblet cells in the intestinal tract of mice subjected to antibiotics treatment (Figure 2h,j), as well as a high-expression of mucin 2 (Figure 2i,k). These findings indicated that Efm has the potential to enhance the anti-tumor efficacy of sorafenib against HCC.

### Efm alters the tumor microenvironment in vivo

**Figure 3.**
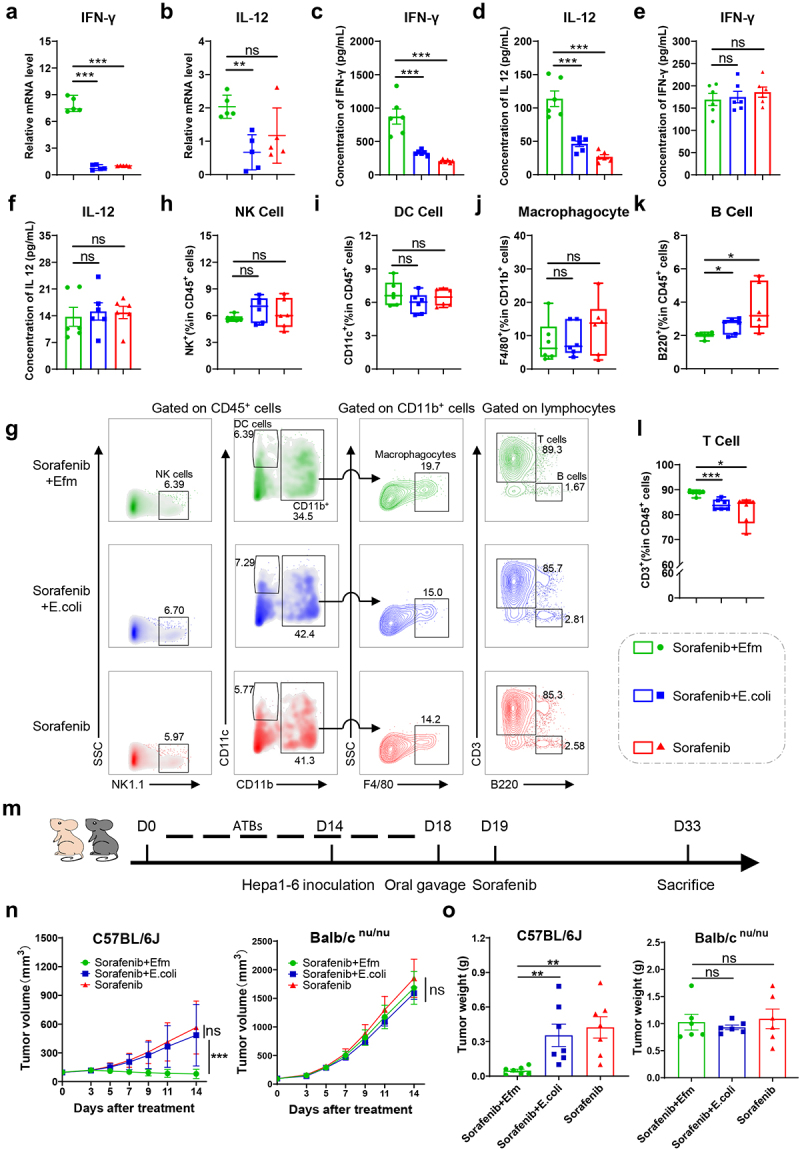
Efm enhanced the efficacy of sorafenib against hepatocellular carcinoma through T cell immunity. (a-b) efm increased the transcription levels of ifn-γ and IL-12 in tumor tissues assessed by PCR. (c-d) efm promoted the expression of ifn-γ and IL-12 in tumor tissues assessed by ELISA. (e-f) efm did not promote the expression of IFN and IL-12 in peripheral blood. (g) The immune cells in the three groups of tumor microenvironment by flow cytometry. (h-l) efm increased the percentage of T cells but not NK cells, DC cells, macrophages, and B cells in the tumor microenvironment. (m) Schematic diagram of the experimental design of bacterial combined with sorafenib in hepa1-6 mouse models in immunocompetent and immunodeficient mice. (n-o) tumor volume and tumor mass of immunocompetent mice and immunodeficient mice after receiving sorafenib in combination with efm, E.Coli or sorafenib alone. Each dot represents a mouse. Error bars represent the SEM. ns *p*>0.05, * *p*<0.05, ** *p*<0.01, *** *p*<0.001.

We performed qRT-PCR to assess the expression of immune factors in the tumor tissues to investigate the molecular mechanism by which Efm enhances the efficacy of sorafenib against HCC. Our results demonstrate no differences in the expression levels of IL-4, IL-6, TNF-α, and TGF-β among the three groups (sorafenib combined with Efm, sorafenib combined with *E.coli*, and sorafenib alone) (Figure S2A). Interestingly however, the expression of IFN-γ and IL-12 in sorafenib combined with Efm was significantly higher than that in the other two groups (Figure 3a,b). Furthermore, the concentration of IFN-γ and IL-12 proteins in tumor tissues of the Efm group were also significantly higher than those of the other two groups as determined by ELISA (Figure 3c,d). However, no significant differences were noted in the levels of IFN-γ and IL-12 in the blood between the three groups (Figure 3e,f), and the concentrations of IL-4, IL-6, TNF-α and TGF-β did not differ among the three groups, either in tumors (Figure S2B) or in blood (Figure S2C).

Therefore, we assessed the immunological effect of Efm in the subcutaneous model of HCC, after two weeks of antibiotic treatment and 10 days of sorafenib combined with bacterial gavage, and specifically analyzed the changes in the proportion of various tumor-infiltrating immune cells such as macrophagocytes, NK cells, DC cells, B cells, and T cells in the TME by flow cytometry (Figure 3g). In the TME, Efm increased the fraction of T cells (Figure 3l) and decreased the sub – population of B cells (Figure 3k), whereas the fraction of macrophagocytes, NK cells and DC cells did not change significantly (Figure 3h–j). Furthermore, changes in immune cells in secondary lymphoid organs (Figure S3A) and peripheral blood (Figure S3C) were detected. The results showed that Efm increased the number of T cells in the spleen but not in the peripheral blood, whereas no change was observed in other immune cells compared with the other two groups (Figure S3B and S3D).

To further investigate whether the anti-tumor effects of Efm enhancing sorafenib activity against HCC were related to T cells, we compared their anti-tumor activities in immunocompetent mice (C57BL/6J) with those in T-cell immunodeficient (Babl/C^nu/nu^) mice using the hepa1-6 HCC model (Figure 3m). As shown by both tumor volume (Figure 3n) and tumor weight (Figure 3o), hepa1-6 tumors grew faster and became larger in Babl/C^nu/nu^ mice, when compared with those in C57BL/6J mice, and Efm failed to enhance the anti-tumor activity of sorafenib in T-cell immunodeficient mice. To assess the effective colonization of the mouse gut by Efm, we first performed FISH analysis of the small intestine and colon mucosa (Figure S2D). We also performed *in vitro* cultures of feces from immunocompetent and immunodeficient mice on enterococcal selective culture plates on the third day after antibiotic administration and Efm supplementation (Figure S2E and S2F). These findings suggest that Efm can enhance immune activation, particularly by increasing the proportion of T cells, which ultimately enhances the anti-tumor efficacy of sorafenib.

### Efm increases IFN-γ^+^CD8^+^ T cells in the tumor microenvironment thereby promoting the anti-tumor activity of sorafenib

**Figure 4.**
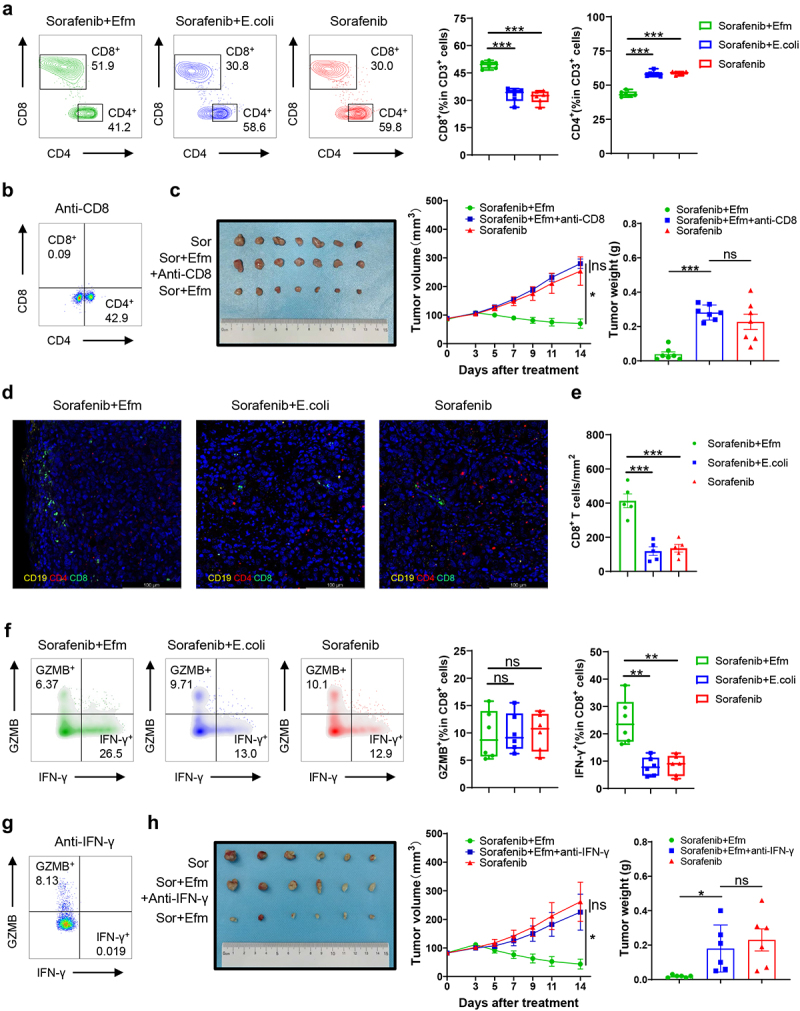
Efm enhanced the anti-tumor ability of sorafenib against hepatocellular carcinoma by increasing the proportion of IFN-γ^+^CD8^+^ T cells in the TME. (a) Efm increased the proportion of CD8 T cells and decreased the proportion of CD4 T cells in the TME. (b) Flow cytometric analysis of the proportion of CD8 T cells in the TME after anti-CD8 antibody application. (c) Anti-CD8 antibody effectively inhibited the enhancement effect of efm on the anti-tumor ability of sorafenib. (d) Representative multiplex immunohistochemistry images of CD4^+^, CD8^+^ T cells and CD19^+^ B cells in tumor tissues with different treatment. (e) CD8^+^ T cell number per mm^2^ was quantified in tumor tissues. (f) Efm increased the proportion of IFN-γ^+^CD8^+^ T cells in the TME. (g) For flow cytometric analysis, the percentage of IFN-γ^+^CD8^+^ T cells in the TME was determined after anti-ifn-γ antibody application. (h) Anti- ifn-γ antibody effectively inhibited the enhancement effect of efm on the anti-tumor ability of sorafenib. Each dot represents a mouse. Error bars represent the SEM. ns *p*>0.05, * *p*<0.05, ** *p*<0.01, *** *p*<0.001. TME, tumor microenvironment.

Next, we isolated lymphocytes from the tumors for flow cytometric analysis, focusing on CD4^+^ and CD8^+^ T cells. The results revealed that Efm induced CD8^+^ T cells but not CD4^+^ T cells (Figure 4a). To evaluate whether the anti-tumor effect of sorafenib promoted by Efm was dependent on CD8^+^ T cell activity, we compared the anti-tumor activity of sorafenib in the presence or absence of CD8^+^ T cells by using an anti-mouse CD8 antibody in the hepa1-6 HCC model and the antibody effectiveness was detected by flow cytometry (Figure 4b). The results showed that the therapeutic efficacy of sorafenib enhanced by Efm was eliminated when an anti-CD8 antibody was used (Figure 4c). Immunohistochemical fluorescence staining of CD4, CD8 and CD19 was performed on subcutaneous tumors in different treatment groups (Figure 4d), and it was found that the number of CD8^+^ T cells in subcutaneous tumors of mice treated with Efm combined with sorafenib was significantly increased (Figure 4e). We then examined the expression of IFN-γ and Granzyme B (GZMB) in CD8^+^ T cells to determine whether IFN-γ^+^CD8^+^ T cells or GZMB^+^CD8^+^ T cells played a major role in this process. The results revealed that tumor-infiltrating CD8 T cells from mice provided with Efm had a higher IFN-γ production compared with that in the other two groups, while GZMB production was similar in all three groups (Figure 4f). Similarly, we used an anti-IFN-γ antibody to inhibit the function of IFN-γ^+^CD8^+^ T cells *in vivo* (Figure 4g) and found that the anti-tumor effect of sorafenib promoted by Efm against HCC was also abrogated (Figure 4h). These findings indicate that Efm increased the fraction of IFN-γ^+^CD8^+^ T cells and promoted the therapeutic efficacy of sorafenib against HCC.

### Exopolysaccharides from efm stimulate IFN-γ^+^CD8^+^ T cells in vitro

**Figure 5.**
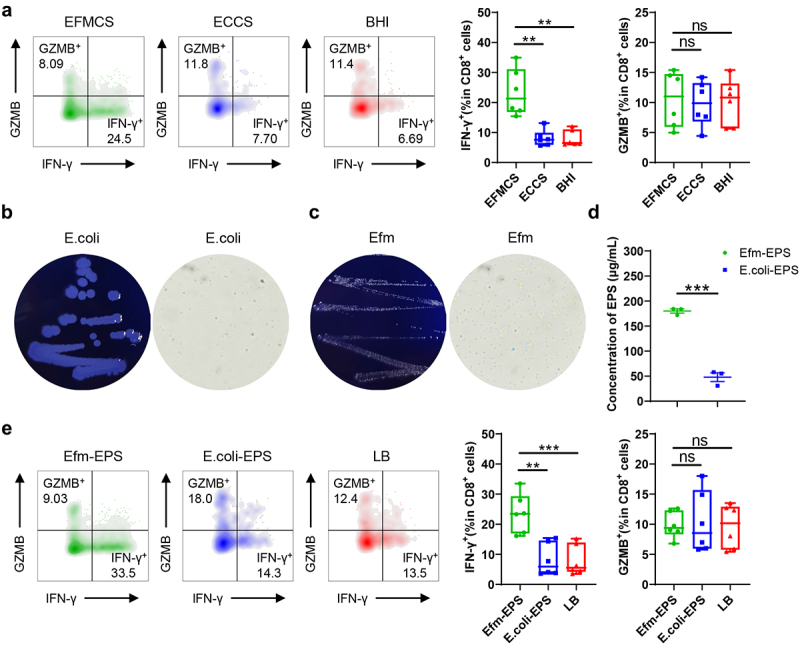
EPS secreted by efm induced IFN-γ^+^CD8^+^ T cells in primary lymphocytes from mouse spleen. (a) EFMCS increased the proportion of IFN-γ^+^CD8^+^ T cells in primary lymphocytes isolated from mouse spleen. (b) Macroscopic and microscopic images of E.Coli cultured in LB plates containing aniline blue. (c) Macroscopic and microscopic images of efm cultured in LB plates containing aniline blue. (d) Quantitative analysis of EPS from E.Coli and efm. (e) Efm-eps increased the proportion of IFN-γ^+^CD8^+^ T cells in primary lymphocytes. Each dot represents a mouse. Error bars represent the SEM. ns *p*>0.05, ** *p*<0.01, *** *p*<0.001. EPS, exopolysaccharides; EFMCS, efm culture supernatants; ECCS, E.Coli culture supernatants; LB, Luria-Bertani; efm-eps, exopolysaccharides of Efm; E.Coli-eps, exopolysaccharides of E.Coli.

To elucidate the mechanism by which Efm increased the number of tumor-infiltrating IFN-γ^+^CD8^+^ T cells, we isolated primary lymphocytes from the spleens of C57BL/6 mice and co-cultured them with Efm and *E. Coli* culture supernatants. Treatment with the culture supernatant of Efm (EFMCS) significantly increased the fraction of IFN-γ^+^CD8^+^ T cells in primary spleen lymphocytes, whereas *E. Coli* culture supernatant (ECCS) and BHI failed to do so. Moreover, there was no change in the proportion of GZMB^+^CD8^+^ T cells in the EFMCS, ECCS or BHI groups (Figure 5a). Subsequently, aniline blue culture plates were used to determine whether Efm and *E. Coli* could produce exopolysaccharides; Efm and *E. Coli* were positive for the production of exopolysaccharides after overnight culture on plates containing aniline blue (Figure 5b,c). Moreover, we assessed the exopolysaccharide-producing capabilities of these two strains and found that the concentration of exopolysaccharides in the EFMCS was significantly higher than that in the ECCS (Figure 5d). To verify if Efm-produced exopolysaccharides were the underlying basis for the rise in the fraction of IFN-γ^+^CD8^+^ T cells, we purified exopolysaccharides from EFMCS and ECCS, and co-cultured exopolysaccharides with primary spleen lymphocytes. Compared to the *E. coli*-EPS treatment and LB treatment groups, the EFM-EPS treatment markedly elevated the fraction of IFN-γ^+^CD8^+^ T cells; however, there was no difference in the proportion of GZMB^+^CD8^+^ T cells among the three groups (Figure 5e). These results suggest that the exopolysaccharides from Efm are capable of increasing the number of IFN-γ^+^CD8^+^ T cells.

### Efm promotes the sensitivity of ferroptosis induced by sorafenib in HCC

**Figure 6.**
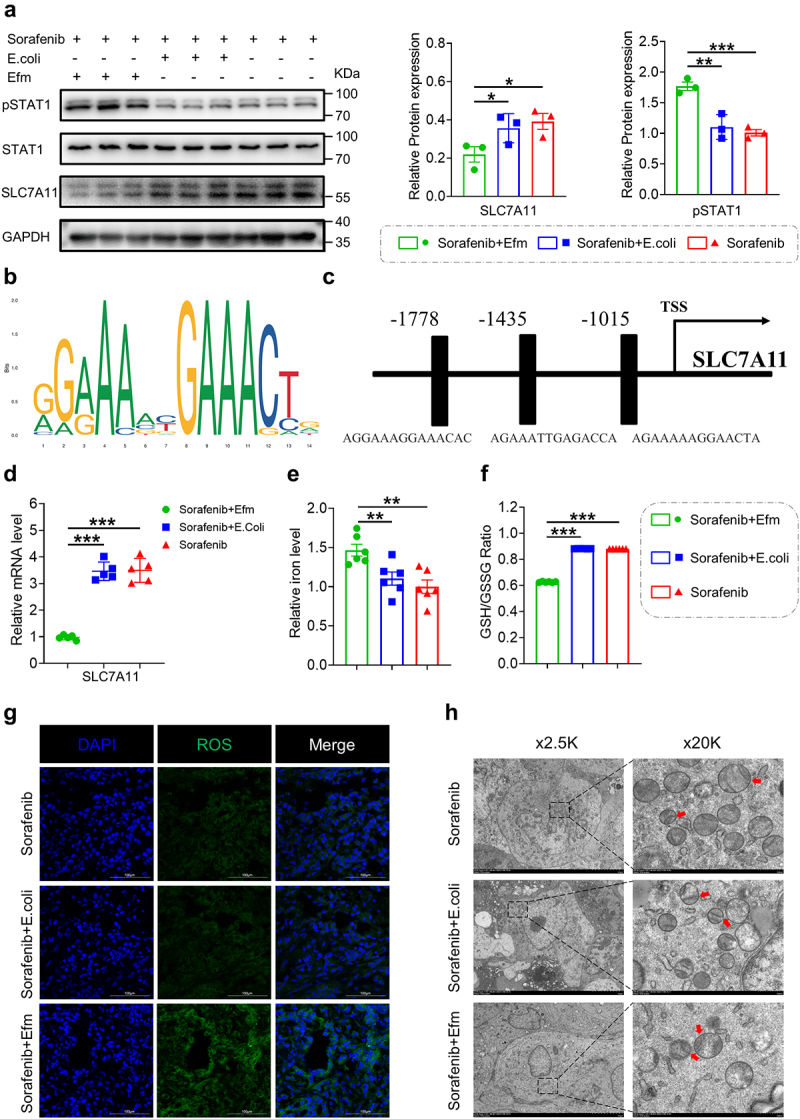
Efm enhanced the ability of sorafenib to induce ferroptosis in hepatocellular carcinoma. (a) The relative protein expression of SLC7A11, STAT1 and pSTAT1 were assessed by western blotting. (b,c) the binding sites between STAT1 and SLC7A11 predicted by JASPAR database. (d) SLC7A11 mRNA levels in tumor tissues with different treatment were detected by qRT-pcr analysis. (e) Representative immunofluorescence staining of total ROS (green) in tumor tissues with different treatment. (f) The morphology of mitochondrial ridges (marked by red arrows) in tumor tissues with different treatment were evaluated by transmission electron microscopy. Each dot represents a mouse. Error bars represent the SEM. ns *p*>0.05, * *p*<0.05, ** *p*<0.01, *** *p*<0.001.

Sorafenib is a ferroptosis inducer, and Wang et al., found that IFN-γ could promote ferroptosis by reducing the SLC7A11 expression through the JAK-STAT1 pathway.^22^ Moreover, SLC7A11 is a sodium-independent cystine-glutamate antiporter that is dependent on chloride, and this system is known as system Xc-. Therefore, we hypothesized that Efm increased the capacity of sorafenib to induce ferroptosis in HCC. To test this hypothesis, the key protein of the system Xc^−^ was detected in tumor tissues after treatment, and the results showed that Efm combined with sorafenib decreased the SLC7A11 protein expression (Figure 6a) in tumors compared to that with *E. Coli* combined with sorafenib and sorafenib alone. In addition, phosphorylated STAT1 expression was significantly increased after Efm supplementation (Figure 6a). At the same time, we found that the expression levels of phosphorylated STAT1 significantly decreased in tumor tissues treated simultaneously with anti-mouse IFN-γ antibody, Efm, and sorafenib compared to the group not treated with anti-mouse IFN-γ antibody (Figure S4). The JASPAR protein database was used to predict binding sites between STAT1 and SLC7A11 (Figure 6b,c). Furthermore, the transcript levels of SLC7A11 in tumors were significantly decreased after combination treatment with Efm and sorafenib (Figure 6d). The intracellular iron levels in tumor cells treated with Efm combined with sorafenib were increased compared to those in the sorafenib group (Figure 6e). Moreover, we evaluated the ratio of reduced and oxidized glutathione in tumor cells and showed that the GSH/GSSG ratio decreased after Efm and sorafenib treatment (Figure 6f). The ROS levels in tumor tissues were assessed by immunofluorescence; combined treatment with Efm and sorafenib resulted in a greater increase in ROS levels than the other two groups (Figure 6g). Transmission electron microscopy revealed the disappearance of mitochondrial ridges in tumor cells treated with Efm combined with sorafenib compared to the other two groups (Figure 6h). These findings suggest that Efm induces the secretion of IFN-γ from CD8^+^ T cells in the TME, thereby markedly augmenting the ability of sorafenib to induce ferroptosis in HCC.

## Discussion

In the current study, we uncovered the crucial components of the gut microbiota implicated in the efficacy of sorafenib, a multikinase inhibitor approved by the FDA as the standard therapy for advanced HCC.^6^ In recent years, an increasing number of studies have demonstrated the impact of gut microbiota on the effectiveness of anticancer drug treatments for malignant tumors, including chemotherapy^23^ and immunotherapy.^24^ However, limited research has been conducted on the relationship between gut microbiota and the efficacy of targeted therapy for unresectable HCC. Herein we showed that two genera of bacteria, *Lachnoclostridium* and *Lachnospira multipara*, as well as *Enterobacter cancerogenus* and Efm were significantly elevated in the intestines of patients with sorafenib-responsive HCC. However, according to the experimental findings, only Efm plays a pivotal role in the anti-tumor efficacy of sorafenib.

With the discovery of an increasing number of disorders linked to an imbalance in the gut microbiota, a new era of intestinal probiotics and microbial therapy is fast approaching.^25,26^ The gut microbiota and their metabolites exert a significant impact on inflammatory signaling pathways, which can directly or indirectly influence the host immune system.^27^ Early studies demonstrated that the gut microbiota can enhance anti-tumor ability by stimulating CD8^+^ T cells,^28^ Th1 cells^29^ and tumor-associated macrophages^30^ in the TME. Moreover, the efficacy of certain chemotherapeutics may be reduced by antibiotic treatment or germ-free mouse models and could be influenced by particular bacterial populations. Importantly, gut -microbiota ameliorates the response of patients to immune checkpoint inhibitors was demonstrated in previously published human studies.^31,32^ Favorable microbiota such as *Clostridiales, Ruminococcaceae, and Faecalibacterium* were able to enhance the function of CD4^+^ T cells and CD8^+^ T cells in the peripheral blood and tumors of melanoma patients.^32^ In addition, we observed an increase in the proportion of T cells within the spleen and TME in hepa1-6 HCC-bearing mice that received a combination of Efm supplementation and sorafenib treatment, whereas the peripheral blood did not. Moreover, *Enterococcus* accumulation in the intestines of patients with anti-PD-1-refractory metastatic melanoma induced by fecal microbiota transplantation (FMT) combined with PD-1 inhibitors led to an increase in intratumoral CD8^+^ T cells and the emergence of tumor necrosis.^32^ In parallel, Tanoue et al., identified 11 beneficial bacterial strains from healthy individuals and demonstrated that their oral administration induced colonic IFN-γ^+^CD8^+^ T cells.^33^ In our present study, oral supplementation with Efm -enhanced the response to sorafenib in hepa1-6 HCC bearing mice by increasing the proportion of IFN-γ^+^CD8^+^ T cells in the TME.

Notably, the principal mechanism by which the gut microbiota regulates anti-tumor immunity is via the production of a multitude of metabolites. These metabolites travel through the intestine to elicit local or systemic anti-tumor immune responses. By increasing the immunogenicity of tumor cells, inosine, a purine metabolite produced by *Akkermansia muciniphila* and *Bifidobacterium pseudolongum*, enhances tumor cell susceptibility to recognition and elimination by cytotoxic T lymphocytes.^34^ In addition, short-chain fatty acids (SCFAs) and butyrate produced by intestinal anaerobic bacteria can enhance the anti-tumor cytotoxicity of CD8^+^ T cells *in vitro* and *in vivo*.^35,36^ Other gut microbiota products such as polysaccharide (PS), outer membrane vesicle (OMV) and peptidoglycan (PG) have been demonstrated to trigger a synergistic anti-tumor immune response. PS secreted by *Leuconostoc mesenteroides* and *Bacteroides fragilis* can be presented by intestinal dendritic cells, prompting CD4^+^ T cells to produce cytokines that promote T cell proliferation.^37^ Luo et al., found that extracellular vesicles from *Akkermansia muciniphila* increased the proportion of GZMB^+^CD8^+^ T and IFN-γ^+^CD8^+^ T cells and induced macrophage recruitment to provoke anti-tumor immunity against prostate cancer.^38^ Recently, the ortholog NlpC/p60 PG hydrolase SagA secreted by Efm was confirmed to promote nucleotide-binding oligomerization domain containing 2 (NOD2) expression and enhance anti-PD-L1 anti-tumor activity.^39^ Interestingly, herein we observed that exopolysaccharides, a distinct type of carbohydrate secreted by Efm, could activate IFN-γ^+^CD8^+^ T cells and enhance IFN-γ expression both *in vitro* and *in vivo*. Previous studies had demonstrated that IFN-γ synthesized by CD8^+^ T cells can impede system Xc- functioning by downregulating SLC7A11 and SLC3A2, two important transporter components of the glutamate-cystine antiporter system Xc^−^, thereby causing ROS accumulation and ferroptosis in fibrosarcoma and melanoma.^22^ These findings are consistent with the results of the present study; although sorafenib is a ferroptosis inducer, the degree of ferroptosis was more severe in mice treated with Efm combined with sorafenib than in those treated with sorafenib alone. This can be attributed to the Efm which increases the number of CD8^+^ T cells that secrete large amounts of IFN-γ.

Although *Enterococci* are potentially pathogenic, they generally exhibit very low virulence, as evidenced by the effective colonization of *Enterococcus* in the intestines of both healthy humans and animals.^40^ Additionally, in this study, patients sensitive to sorafenib with a high proportion of Efm did not have any adverse intestinal reactions, and mice that were orally supplemented with Efm did not develop adverse effects, including diarrhea, bloody stool and weight loss (data not shown). It is important to thoroughly determine whether Efm should be the primary probiotic used in conjunction with sorafenib for the treatment of advanced HCC. Further research is warranted to corroborate this therapeutic possibility.

**Figure 7.**
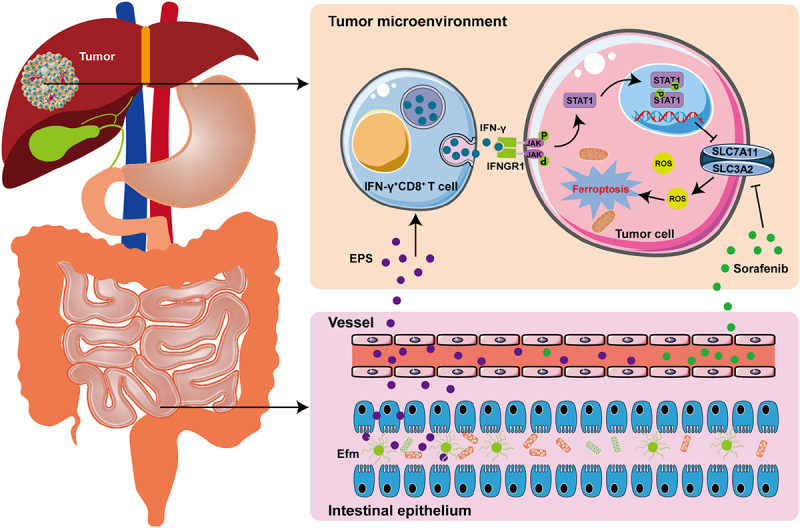
Schematic illustration of EPS secretion by efm to induce IFN-γ^+^CD8^+^ T cells to promote ferroptosis induced by sorafenib in hepatocellular carcinoma. The EPS secreted by efm in the intestine enters the tumor microenvironment of HCC and prompts IFN-γ^+^CD8^+^ T cells to secrete ifn-γ. ifn-γ enters HCC cells to activate the JAK-STAT1 signaling pathway and produce phosphorylated STAT1, which acts as a transcription factor to inhibit the transcription of SLC7A11, thereby increasing intracellular ROS and inducing ferroptosis in HCC cells.

In summary, our current study is the first to reveal that critical gut microbiota is involved in the anti-tumor effect of sorafenib in patients with advanced HCC. Of course, there are still some limitations in our study. For example, in this study, we used a subcutaneous model instead of a primary liver cancer model or an orthotropic model. Our results demonstrated significant enrichment of Efm in the intestines of patients with advanced HCC who responded favorably to sorafenib treatment. Additionally, our experiments on hepa1-6 HCC bearing mice showed that combining Efm with sorafenib resulted in enhanced anti-tumor activity by increasing the fraction of tumor-infiltrating IFN-γ^+^CD8^+^ T cells. Furthermore, Efm stimulated intratumoral IFN-γ^+^CD8^+^ T cells through secreted exopolysaccharides to produce IFN-γ, which cooperated with sorafenib to trigger ferroptosis in HCC (Figure 7). These findings reveal the mechanism by which the gut microbiota influences the anti-tumor ability of sorafenib in advanced HCC. This study identified a novel strategy for the synergistic combination of intestinal probiotics and sorafenib as a potent therapy to markedly enhance treatment efficacy in patients with advanced HCC.

## Material and methods

### Study participants

Twenty advanced HCC patients who received first-line sorafenib therapy were recruited between January 2020 and January 2022 at the First affiliated Hospital of Wenzhou Medical University. The diagnosis of advanced HCC depends on hematological tests, iconography examination and pathological biopsy. Each recruited patient met the following strict inclusion criteria: 1) Diagnosis of advanced primary HCC without an opportunity for surgical resection. 2) No history of hypertension, diabetes, chronic renal failure, metabolic diseases, or other malignant tumors. 3) No anti-tumor therapy was administered before this treatment. 4) No antibiotics or probiotics were used during therapy. 5) Able to tolerate adverse reactions to sorafenib and have regular follow-ups. The patients in this study were all from the local Wenzhou population with similar dietary patterns. During the treatment, all patients were required to maintain their dietary patterns. Hematological tests and iconographic examinations were used to assess the efficacy of sorafenib treatment according to RECIST 1.1 standards every three months after receiving sorafenib treatment. We categorized the patients into two cohorts based on treatment outcomes: responders (R, *n* = 9) and non-responders (NR, *n* = 11). Patients who achieved a complete or partial response or who maintained stable disease for >6 months were classified as responders, whereas those who experienced progressive disease were characterized as non-responders. All patients were monitored every three months, and the most recent observation was conducted in September 2022. All patients signed a written informed consent form before being recruited for the study, which was approved by the Ethics Committee of the First Affiliated Hospital of Wenzhou Medical University.

### Fecal sample collection, 16S rRNA sequencing and analysis

Fresh fecal specimens were collected during the patient’s hospital stay for hematology and imaging evaluations and promptly preserved at − 80°C before further analysis. The feces of responders at the last clinic visit and the feces of non-responders before the treatment plan was changed were used for 16S rRNA sequencing. Bacterial diversity and taxonomic analyses were performed using R software and GraphPad Prism 8. The chao1 index was employed to indicate alpha diversity, whereas principal coordinate analysis (PCoA) was utilized for beta diversity to assess the dissimilarity in fecal microbial composition between the two groups. Linear discriminant analysis (LDA) effect size (LEfSe) and Cladogram of taxa were used to determine the differentially abundant microbial taxa in the fecal samples.

### Cell lines, bacteria and sorafenib

The mouse HCC cell line hepa1-6 was obtained from the China Center for Type Culture collection (Wuhan, Hubei, China) and cultured in high-glucose DMEM (Gibco, C11995500BT) containing 10% fetal bovine serum (Gibco, 10099141C) and 1% Penicillin-Streptomycin (Solarbio, P1400; Beijing, China) at 37°C under a humidified atmosphere of 5% CO_2_. *Lachnoclostridium bouchesdurhonense, Lachnoclostridium massiliosenegalense, Lachnoclostridium touaregense, Lachnospira multipara, Lachnospira eligens, Enterobacter cancerogenus and Enterococcus faecium* were acquired from DSMZ (German Collection of Microorganisms and Cell Cultures GmbH, Braunschweig, Germany). All bacteria were cultured under aerobic or anaerobic conditions in a suitable growth medium at 37°C with 5% CO_2_. Sorafenib for patient treatment was purchased from Bayer Pharma AG (Bayer, Leverkusen, Germany), and the sorafenib used to treat mice was purchased from MedChemExpress (MCE, HY-10201, New Jersey, USA).

### In vivo anti-tumor treatment of hepa1-6 tumor models

CAnN.Cg-Foxn1nu/CrlCrlj (Balb/c^nu/nu^) and C57BL/6J mice were obtained from Hangzhou Medical College (Hangzhou, China). All mice were housed in specific-pathogen-free conditions controlled at 21–25°C,12:12 light/dark cycle. Male mice, aged 6–10 weeks, were subcutaneously injected with Hepa1-6 cells (3 × 10^6^) in the right flank. Once the tumor volume reached 100 mm^3^, the mice were randomly divided into different treatment groups and sorafenib was administered (dissolved in Cremophor EL/ethanol, 30 mg/kg) by oral gavage once daily for 14 days. *Lachnoclostridium bouchesdurhonense, Lachnoclostridium massiliosenegalense, Lachnoclostridium touaregense, Lachnospira multipara, Lachnospira eligens* were grown on sheep’s blood agar plates (Bio-Kont, Wenzhou, China) for 24 h at 37°C under aerobic conditions. *Enterobacter cancerogenus and Enterococcus faecium* were inoculated onto sheep’s blood agar plates and incubated aerobically at 37°C and 5% CO_2_ for 24 hr. Bacteria were then harvested from the agar medium and suspended in sterile saline solution. The bacterial concentration was determined by measuring the optical density at 600 nm, with an OD value of 1, corresponding to approximately 1 × 10^9^ colony-forming units (CFU)/ml. In the experiment involving the combined treatment with sorafenib and bacteria, the mice were orally administered 1 × 10^9^ CFUs and then treated with sorafenib the following day. Bacteria were administered via oral gavage twice weekly. For CD8^+^ T cell or IFN-γ^+^CD8^+^ T cell depletion experiment, mice were intraperitoneally injected with anti-mouse CD8 antibody (Bio X Cell; YTS 169.4) and control IgG (Bio X Cell; LTF- 2) or anti-mouse IFN-γ antibody (Bio X Cell; R4-6A2) and control IgG (Bio X Cell; HRPN). Three days after the injection of the antibody, the mice were orally administered the bacteria and then received sorafenib treatment the following day. Each mouse was injected with 200 μg of anti-mouse CD8 antibody or control IgG, twice a week.

The tumor volume was measured using a caliper every two days. The tumor volume was calculated as follows: Tumor volume (mm^3^) = 0.523× (length [mm] × width^2^[mm^2^]). The calculation formula of ΔT/C (% of control for Δ growth) was as follows: ΔT/ΔC × 100%, where ΔT and ΔC are the values of tumor volume changes in the treatment group and control group, respectively. All animal experiments in the current study were approved by the Animal Ethics Committee of the First Affiliated Hospital of Wenzhou Medical University and were implemented in accordance with the ARRIVE guidelines.

### ELISA

Each mouse was subjected to tumor tissue isolation and weight assessment on the 9^th^ day of sorafenib treatment. We added 1 ml of cold MPER lysis buffer containing protease and phosphatase inhibitors to every 100 mg of tumor tissue, homogenized on ice for 30 seconds, and finally centrifuged at 18,000 g for 15 minutes to remove cell debris and lipids, after which the supernatant was collected. The levels of cytokines IL-4 (431104), IL-6 (431304), IL12 (433604), IFN-γ (430804) and TNF-α (430904) in tumor tissues and serum were measured by ELISA kits (BioLegend, San Francisco, CA, USA) following the kit’s instructions. The levels of TGF-β were determined by ELISA kits (Thermo Fisher, 88-8350-22; Waltham, MA, USA). All cytokine data were obtained from at least two independent ELISA tests.

### ATB treatments and colony forming unit (CFU) analysis

Animals were orally gavaged with antibiotic (ATB) mixture containing ampicillin (1 g/L, Absin, abs9224), neomycin (1 g/L, Absin, abs816418), metronidazole (0.5 g/L, Absin, abs47047848) and vancomycin (0.5 g/L, Absin, abs815996) twice a day for 14 days before bacterial inoculation and sorafenib treatment. The antibiotic activity was assessed by culturing feces on days 3 and 10 of antibiotic gavage. Fresh feces were collected from the mice by abdominal massage, weighed, and resuspended in brain heart infusion (BHI)+15% glycerol. Next, the fecal suspension was serially diluted and cultured on sheep’s blood agar plates (Bio-kont, Wenzhou, China) for 24 hours at 37°C under aerobic conditions. Similarly, Efm efficient colonization was evaluated by fecal culture onto selective enterococcosel agar plates (Hopebio, China) for 24–48 hours at 37°C under aerobic conditions. Colonies were counted manually after 24–48 hours of incubation until individual colonies were formed.

### Single cell isolation and flow cytometry

Blood, spleen and tumors of C57BL/6J tumor-bearing mice were harvested nine days after bacteria inoculation combined with sorafenib or sorafenib alone. Blood was collected in an EDTA anticoagulant tube, erythrocytes were lysed using by Ammonium-Chloride-Potassium (ACK) lysis buffer (Yeasen, 40401ES60), centrifuged at 400×g for 5 minutes, and the supernatant was discarded. The spleens were gently mashed using the back of a syringe, and the cells were filtered through a 70 μm cell strainer. Then, using the ACK lysis buffer to lyse and eliminate erythrocytes, a percoll gradient (Yeasen, 40501ES60) was used to purify lymphocytes, and the pelleted cells were resuspended in 3 ml solution of 40% percoll solution and gently added to the top of 3 ml 70% percoll solution. Lymphocytes between the 40% and 70% percoll layers were aspirated using a dropper, after which centrifugation at 400×g for 30 min was performed. Lymphocytes were prepared for flow cytometric analysis. Tumors were collected and manually isolated from each mouse and cut into small pieces (~1 mm), then digested in RPMI-1640 medium (Gibco, C11875500BT) containing Liberase (2 mg/mL, Roche 05,401,020,001; Basel, Switzerland) and DNase I (10 mg/mL, Roche 10,104,159,001; Basel, Switzerland) shaking at 37°C for 30 min. The mixture was passed through a 70 μm cell strainer followed by ACK lysis buffer and percoll gradient to isolate lymphocytes, which were free of erythrocytes. All purified lymphocytes from blood, spleen and tumors were incubated with anti-mouse CD16/CD32 (BioLegend 101,320) for 15 min at 4°C before membrane staining, for intracellular staining of IFN-γ and GZMB, all lymphocytes were stimulated with PMA (50 ng/ml, MCE, HY-18739) and ionomycin (1ug/ml, MCE, HY-13434) for 4 h and incubated with Brefeldin A (10 μg/ml, MCE, HY-16592) for 2 h in RPMI-1640 medium containing 10% FBS. To evaluate the effect of the bacterial culture supernatant and exopolysaccharides on primary lymphocytes, 10% Efm culture supernatant (EFMCS), *E. Coli* culture supernatant (ECCS), BHI, and exopolysaccharides solution were added to the growth medium. Cytofix/Cytoperm (BD biosciences 554,722; Franklin Lakes, New Jersey, USA) was used to fix and permeabilize the cells. Anti-mouse antibodies for CD45 (Clone 104, 109806), CD3 (Clone 17A2, 100236), CD4 (Clone GK1.5, 100432), CD8 (Clone 53–6.7, 100708), CD11b (Clone M1/70, 101212), CD11c (Clone N418, 117308), F4/80 (Clone BM8, 123114), NK1.1 (Clone PK136, 108714), IFN-γ (Clone XMG1.2, 505826), GZMB (Clone QA18A28, 396414), Zombie UV^TM^ Fixable Viability Kit (423107) were obtained from BioLegend. The samples were resuspended in FACS buffer and analyzed using an LSRFortessa X-20 (BD Biosciences). The FlowJo software (v10, BD Biosciences) was used for data analysis.

### Immunohistochemistry, alcian blue & nuclear fast red staining

The small intestine and colon tissues of the mice were separated and fixed in 4% paraformaldehyde solution for 24 hours. The tissues were dehydrated through a series of alcohol and toluene concentration gradients, and then embedded in paraffin. The tissue sections, which were 5 μm thick, were affixed to glass slides and incubated at 60°C for 2 hours and were subsequently dewaxed in an environmentally friendly dip-wax transparent solution for 10 min. Finally, the sections were dehydrated using a series of various concentrations of alcohol and distilled water. Endogenous peroxidases in tissues were inactivated using 3% hydrogen peroxide for 10 min at room temperature (RT). Antigen retrieval was carried out by heating a citric acid solution in a microwave for 15 minutes, followed by cooling to RT and blocking with 5% BSA in PBS for 1 h at 37°C. Finally, primary antibody of Muc-2 (1:500, Servicebio, GB11344; Wuhan, Hubei, China) was applied overnight at 4°C. The secondary antibody (1:200, Biosharp, BL003A; Hefei, Anhui, China) was incubated for 1 hour and the biotin-free peroxidase system of detection with 3,3’-diaminobenzidine (DAB) (Servicebio, G1212-200T; Wuhan, Hubei, China) as a chromogen was used to evaluate Muc-2 positive signal. Nuclear counterstaining was performed using hematoxylin followed by dehydration. Alcian Blue and Nuclear Fast Red staining were used to quantify the number of goblet cells in the small intestine and colon. After the tissue sections were dewaxed and dehydrated, Alcian blue solution was added to the slides for 30 min at RT, after three washes, the nuclei were stained with the nuclear fast red for 3 min, then rinsed with running water. The sections were dehydrated three times with ethanol for 5 min each, and then rendered transparent with xylene for 5 min before being sealed with neutral gum. Images were captured using a LEICA DM750 microscope (Leica, Wetzlar, Germany), Muc-2 positive area was analyzed using ImageJ software. For goblet cell counting, the number of Alcian Blue-positive cells in five nonadjacent villi or crypts was counted for each sample. For Muc-2 positive area and goblet cell number statistics, six slides per group were calculated.

### Multiplex immunohistochemistry assay and analysis

Fresh tumor tissues were isolated from the mice and fixed in 4% paraformaldehyde for 24 hours. Dehydration was performed with different concentrations of alcohol and toluene, and then embedded in paraffin. 5 μm-thick tumor tissue sections were used for multiplex immunohistochemistry analysis. All slices were incubated for 2 hours at 60°C, dewaxed with dewaxing solution, and dehydrated in alcohol at different concentrations. Endogenous peroxidase inactivation, antigen retrieval, and blocking were performed using immunohistochemistry. CD8^+^ T cells were visualized using a CD8 antibody (1:500, Invitrogen, MA1–10301), secondary antibody (1:200, Biosharp, BL001A) and fluorescein FITC-Tyramide (Servicebio, G1222). The specific steps were as follows: primary antibody of CD8 was applied for 2 hours at 37°C, washed three times with PBS, secondary antibody was incubated for 1 hour at 37°C, washed three times and then added to TSA working solution containing FITC (1 ml TBST, 1ul 3%H_2_O_2_, 2ul FITC-Tyramide) in a dark room. Next, repeated the inactivation of endogenous peroxidases, antigen retrieval and antigen blocking, visualization of CD4^+^ T cells using CD4 antibody (1:500, Proteintech 19,068–1-AP), secondary antibody (1:200, Biosharp, BL003A) and fluorescent iF647-Tyramide (Servicebio, G1232). Repeated the inactivation of endogenous peroxidases, antigen retrieval and antigen blocking again. Visualization of CD19^+^ B cells was performed using CD19 antibody (1:500, Santa cruz, sc -373,897), secondary antibody (1:200, Biosharp, BL051A) and fluorescent CY3-Tyramide (Servicebio, G1223), and nuclei were visualized with the antifade mounting medium with DAPI (Solarbio, S2110). Images were obtained using a confocal microscope (Nikon C2si, Japan).

### Immunofluorescence and fluorescence in situ hybridization

Fresh tumor tissues were isolated from mice, embedded in OCT (Sakura Finetek, Nagano, Japan) and frozen. The 5 μm-thick tissue sections were placed on glass slides and fixed with 4% paraformaldehyde for 10 min, blocked with 5% BSA/PBS for 1 hour at 37°C. The Reactive Oxygen Species Assay Kit (Beyotime, S0033S) was used to assess the total ROS in tumor tissue, fluorescence probe DCFH-DA was added to glass slides at a concentration of 1/1000 and incubated for 1 hour at 37°C. After three washes, the nuclei were stained with antifade mounting medium containing DAPI (Solarbio, S2110). For fluorescence in situ hybridization, Fresh small intestine and colon tissues were isolated from C57 mice on the third day after ATBs administration and Efm supplementation. These tissues were fixed in 4% paraformaldehyde for 24 hours, dehydrated through different concentration gradients of alcohol and toluene and embedded in paraffin; next, 5 μm-thick sections of small intestine and colon tissue were placed on glass slides and incubated at 60°C for 2 h followed by being dewaxed and dehydrated with a series of different concentrations of alcohol. Slides were pre-treated with proteinase K (10ug/ml, Beyotime, ST535) for 30 min at 37°C, and hybridization was performed overnight at 37°C by applying a general bacterial probe (TTCACACAATCGTAACTTCC, Servicebio) labeled with the fluorophore 5-FAM at a concentration of 5 ng/µl, specific for the genus Enterococcus. Hybridization and wash buffers were used to remove nonspecific probe binding, and an antifade mounting medium with DAPI was used to counterstain the nuclei. Images were scanned with a confocal laser microscope (Nikon C2si, Japan).

### Quantitative real-time PCR

The hepa1-6 tumor tissue was isolated on the ninth day from mice inoculated with bacteria and treated with sorafenib; total RNA was extracted from the tumor tissue using the RNAeasy^TM^ Animal RNA Isolation Kit (Beyotime, R0027; Shanghai, China). After determining the RNA concentration, cDNA was synthesized using PrimeScript^TM^ RT Master Mix (TaKaRa, RR036A; Kusatsu, Shiga, Japan). The SYBR-Green Master Mix kit was used to perform qRT-PCR on a PCR Detection System (Applied Biosystems, ABI Prism 7500 system). β-actin was used as the internal standard. The primers for IL-4 are:

**Table:** 

primers	Forward	Reverse
IL-4	TACCAGGAGCCATATCCACGGATG	TGTGGTGTTCTTCGTTGCTGTGAG
IL-6	TAGTCCTTCCTACCCCAATTTCC	TTGGTCCTTAGCCACTCCTTC
IL-12	GACCTGTTTACCACTGGAACTA	GATCTGCTGATGGTTGTGATTC
IFN-γ	CTTGAAAGACAATCAGGCCATC	CTTGGCAATACTCATGAATGCA
TGF-β	CCAGATCCTGTCCAAACTAAGG	CTCTTTAGCATAGTAGTCCGCT
TNF-α	CCCTCACACTCAGATCATCTTCT	GCTACGACGTGGGCTACAG
SLC7A11	TTACCAGCTTTTGTACGAGTCT	GTGAGCTTGCAAAAGGTTAAGA

### Western blot analysis

Appropriate fresh tissue was homogenized in a lysis buffer using a homogenizer, and a BCA Protein Assay kit (Beyotime, P0011; Shanghai, China) was used to calculate total protein concentrations. Equal amounts of protein lysates were resolved on SDS-PAGE gels and transferred to PVDF membranes. After blocking for 15 min with a quickblock buffer (Beyotime, P0235; Shanghai, China), the primary antibody was used upon an overnight incubation at 4°C, including Anti -Interferon γ Rabbit pAb (1:500, Servicebio, GB11107–1), Anti-xCT Rabbit monoclonal antibody (1:1000, Abcam, ab175186), anti-STAT1 Rabbit polyclonal antibody (1:2000, Proteintech 10,144–2-AP), anti-pSTAT1 Rabbit polyclonal antibody (1:1000, Proteintech 28,977–1-AP)and Anti-GAPDH Rabbit monoclonal antibody (1:5000, Abcam, ab181602). Protein bands were incubated with secondary antibodies for 1 h at RT and visualized with HRP (Thermo fisher 34,096). The images of positive protein bands were captured using an imaging system (Aplegen, Omega lum w; Vista, California, United States).

### Identification and purification of exopolysaccharide production

Efm and *E. coli* were streaked and cultured overnight on LB plates containing aniline blue (10 g/L, tryptone, 5 g/L, yeast extract, 10 g/L, sodium chloride, 0.5 g/L, aniline blue, 1.5% w/v, agar). The appropriate number of Efm and E. coli colonies were scraped out with a sterile cotton stick and resuspended in PBS. PBS containing the colonies was dropped onto a glass slide to make a bacterial smear and the color of the bacterial was observed under a microscope at 1000× magnification. For the identification of exopolysaccharides, the sulfate-anthrone method was used to determine the production of exopolysaccharides in the supernatant of Efm and *E. Coli* cultures. For exopolysaccharide purification, Efm and *E. Coli* were cultured in LB sugar producing medium (10 g/L, tryptone, 5 g/L, yeast extract, 10 g, sodium chloride, 0.5 g/L, sucrose) at 180 g/min for 40 hours at 37°C. Thereafter, compound proteinase (10 U/ml, Solarbio, C8800; Beijing, China) was added to the medium and digested for 4 hours at 50°C, then the compound protease was inactivated by heating for 20 min in a 100°C water bath followed by centrifugation 20 min at 3200 g/min to remove the bacteria and proteins. The supernatant was retained and twice the volume of 95% cold alcohol was added and kept at 4°C overnight in the dark. The solution was centrifuged at 3200 g/min for 20 min again, the supernatant was removed, the exopolysaccharide was dissolved with 0.2 ml of distilled water, and twice the volume of 95% cold alcohol was added and kept at 4°C overnight in the dark. The supernatant was removed by centrifugation, and the exopolysaccharide was dissolved in 0.2 ml distilled water for concentration detection using the sulfate-anthrone method.

### Detection of intracellular iron and GSH/GSSG ratio

The method of isolating tumor cells is similar to that of isolating lymphocytes, both of which use density gradient centrifugation. However, after digestion, filtration, and elimination of erythrocytes in tumor tissue, 80% percoll was used for gradient centrifugation at 400 g for 30 minutes, the precipitated tumor cells were washed with PBS 3 times for the intracellular iron and glutathione detection. Intracellular iron of tumor cells was detected using a Ferrous Iron Colorimetric Assay Kit (Elabscience, E-BC-K773-M; Wuhan, China), take 1 × 10^6^ tumor cells obtained by centrifugation, then, cells were homogenized under RlPA buffer (strong, 100 uL) mixed with extracting solution. Next, the relative iron level was assessed using a kit according to the instruction. Finally, OD value was detected using a microplate reader at an absorbance of 593 nm. Reduced glutathione (GSH) and oxidized glutathione disulfide (GSSG) in tumor tissue were detected using a GSH and GSSG assay kit (Beyotime, S0053; Shanghai, China). Briefly, take 1 × 10^6^ tumor cells obtained by centrifugation and add three times the volume of protein removal agent M solution, then, lysed the tumor cells by two cycles of freezing and thawing using liquid nitrogen and a 37°C water bath. The samples were centrifuged at 1000 g at 4°C for 10 minutes, and the supernatant was collected for detection of total glutathione and GSSG. GSH was acquired by subtracting GSSG from total glutathione. Total glutathione and GSSG were detected using a microplate reader at an absorbance of 412 nm.

### Statistical analysis

Statistical analysis was performed with prism 8 (GraphPad, San Diego, CA, USA) and R. All data were presented as mean±SEM. Comparisons between two or multiple groups were performed using the unpaired t-test and one-way ANOVA. All reported tests were two-tailed and were considered statistically significant at *p* < 0.05.

## Abbreviations

ACK: Ammonium-Chloride-Potassium
BHI: Brain-Heart Infusion
CFU: colony forming unit
DAB: detection with 3,3’-diaminobenzidine
ECCS: E.coli culture supernatant
EFMCS: Efm culture supernatant
Efm: Enterobacter faecium
EPS: exopolysaccharides
FLT-3: FMS-like tyrosine kinase 3
FMT: fecal microbiota transplantation
GSH: glutathione
GSSG: oxidized glutathione disulfide
GZMB: Granzyme B
HCC: hepatocellular carcinoma
LDA: Linear discriminant analysis
LEfSe: Linear discriminant analysis effect size
MTA: molecular-targeting agent
OMV: outer membrane vesicle
OTUs: operational taxonomic units
PCoA: principal coordinates analysis
PDGFRβ: platelet-derived growth factor receptor-β
PG: peptidoglycan
PS: polysaccharide
TME: tumor microenvironment
VEGFRs: vascular endothelial growth factor receptors

## Supplementary Material

Supplemental figures new.docx

## Data Availability

All relevant data are available from the authors upon request.
