# Supplementary material for: Synergistic activity of Enterococcus Faecium-induced ferroptosis via expansion of IFN-γ+CD8+ T cell population in advanced hepatocellular carcinoma treated with sorafenib
Source: Gut Microbes. 2024 Oct 1;16(1):2410474. doi: 10.1080/19490976.2024.2410474 (PMC11445893; doi:10.1080/19490976.2024.2410474)
Supplement: Supplemental figures new.docx [file KGMI_A_2410474_SM3688.docx]

**Supplementary figures and figure legends**

**
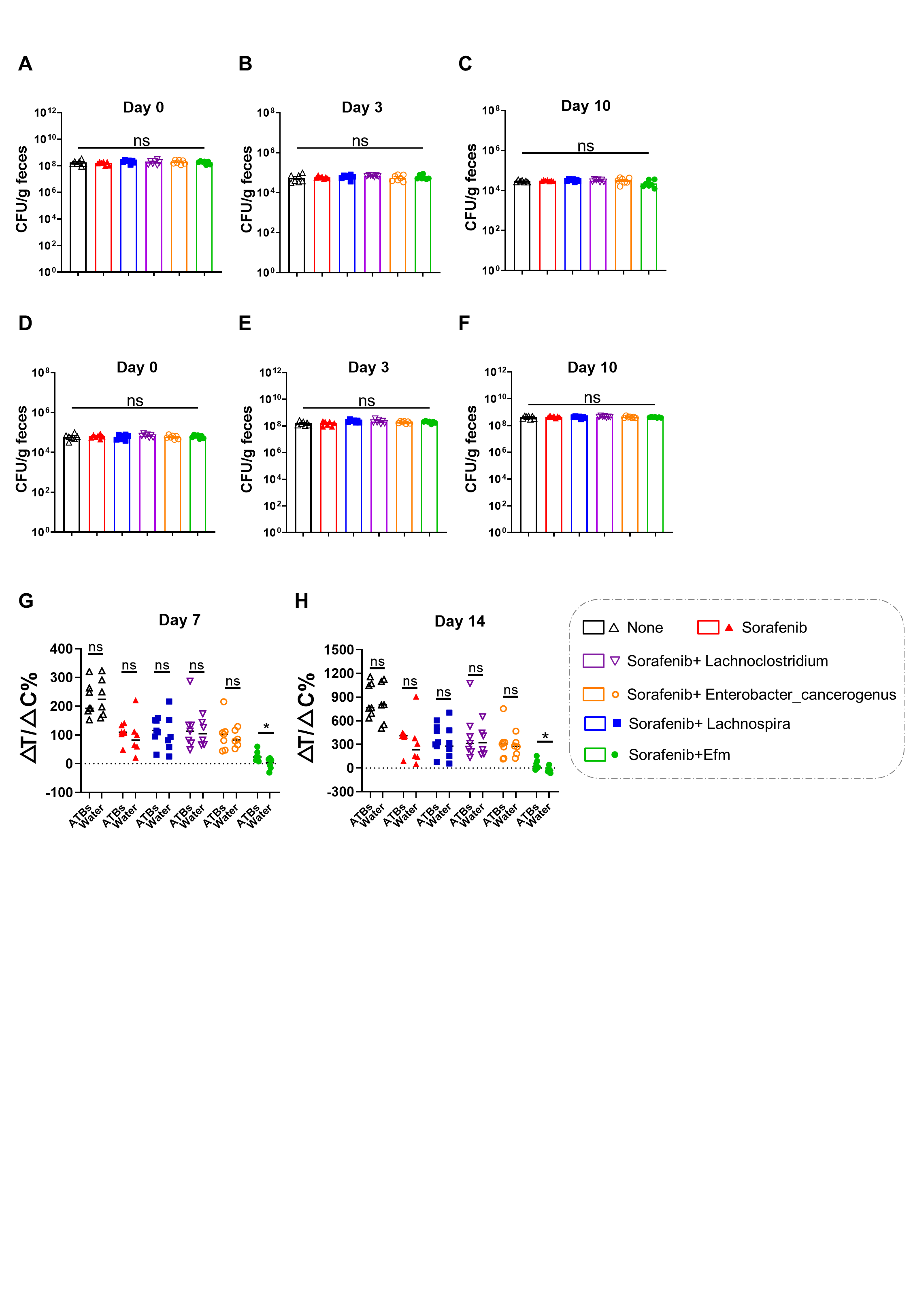
Figure S1**

**Fig S1.** Fecal microbiota culture after treatment with ATBs and supplementation with microbiota, and antitumor activities shown as ΔT/C values. (A-C) CFU analysis of fecal microbiota in mice treated with ATBs for 0 day, three days and ten days. (D-F) CFU analysis of fecal microbiota in mice supplementation with various microbiota for 0 day, three days and ten days after ATBs treatment. (G-H) Antitumor activities shown as ΔT/C values of sorafenib combined with different bacterial in Hepa1- 6 mice tumor models at days 7 and 14. Each dot represents a mouse. Error bars represent the SEM. ns P>0.05, * P<0.05. ATBs, antibiotics; CFU, colony-forming unit.


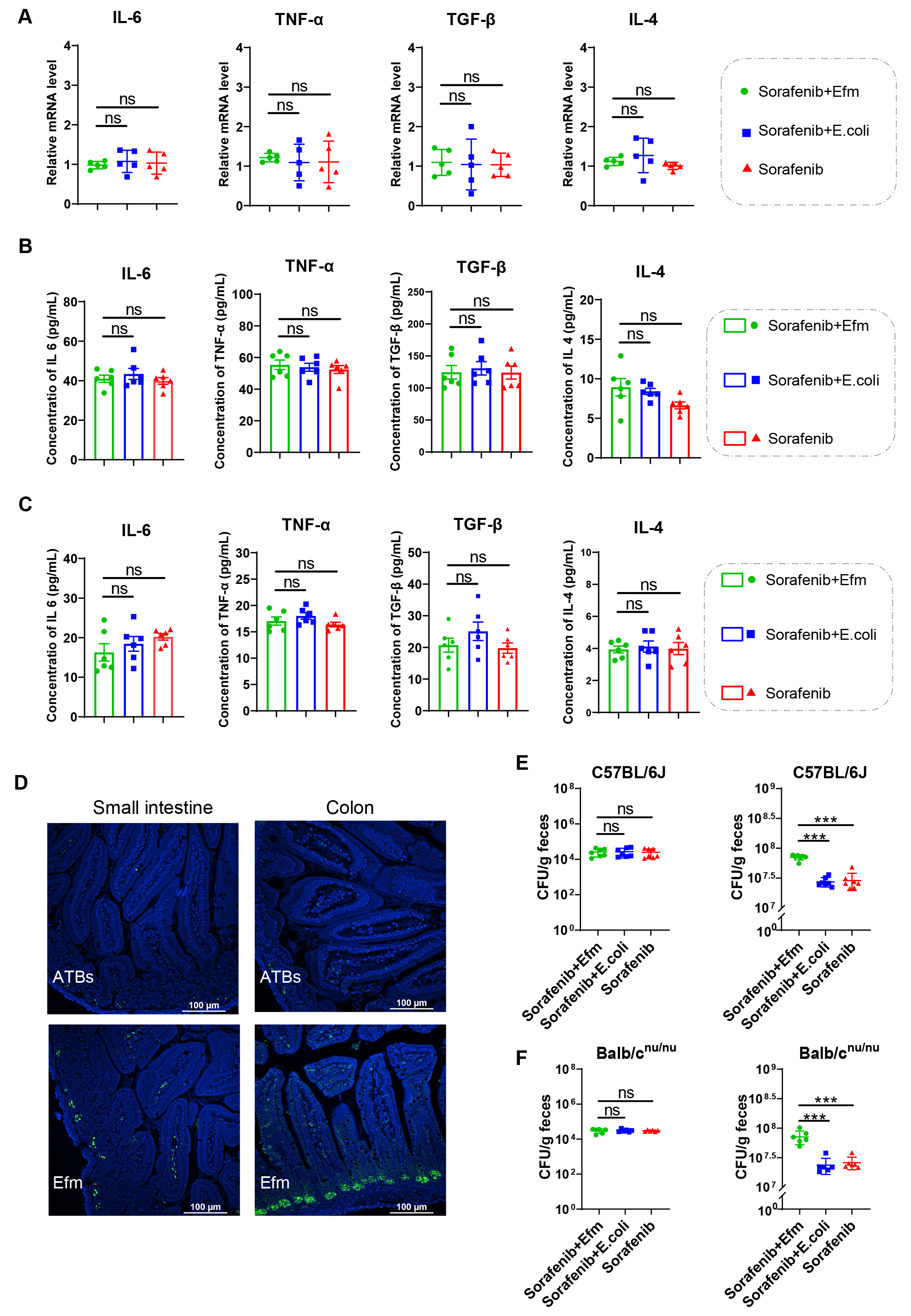
**Figure S2**

**Fig S2.** Effects of Efm on immune factors in tumor microenvironment and peripheral blood of mice treated with sorafenib, and Effective colonization and number of Efm in the intestine of mice after antibiotic administration and Efm supplementation. (A) Efm failed to alter IL-6, TNF-α, TGF-β and IL-4 transcript levels in the tumor tissues assessed by PCR. (B) Efm failed to alter the expression of IL-6, TNF-α, TGF-β and IL-4 in the tumor tissues assessed by ELISA. (C) Efm failed to alter the expression of IL-6, TNF-α, TGF-β and IL-4 in peripheral blood. Each dot represents a mouse. (D) FISH analyses of Efm in the small intestinal and colon mucosa on the third day after antibiotic administration and Efm supplementation. (E) Quantity of Efm in feces of immunocompetent mice on the third day after antibiotic administration and Efm supplementation. (F) Quantity of Efm in feces of immunodeficient mice on the third day after antibiotic administration and Efm supplementation. Each dot represents a mouse. Error bars represent the SEM. ns P>0.05, *** P<0.001.


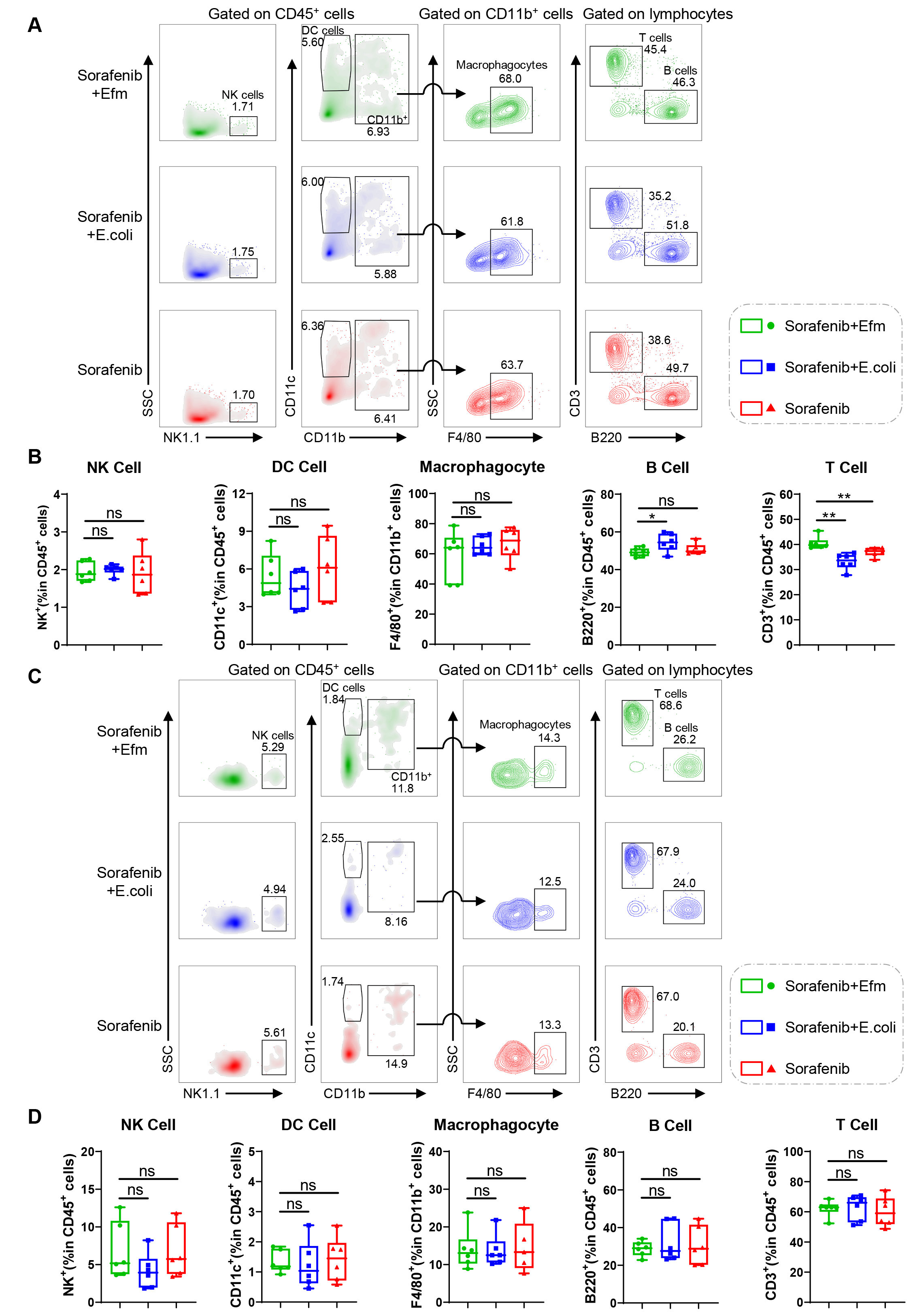
**Figure S3**

**Fig S3**. Effect of Efm on immune cells in spleen and blood in sorafenib treated mice. (A) The immune cells in the spleen of mice treated with Efm and sorafenib by flow cytometry. (B) Efm increased the proportion of T cells in the spleen in sorafenib treated mice. (C) The immune cells in peripheral blood of mice treated with Efm and sorafenib by flow cytometry. (D) Efm did not change the proportion of immune cells in peripheral blood in sorafenib treated mice. Error bars represent the SEM. ns P>0.05, * P<0.05, ** P<0.01.

**
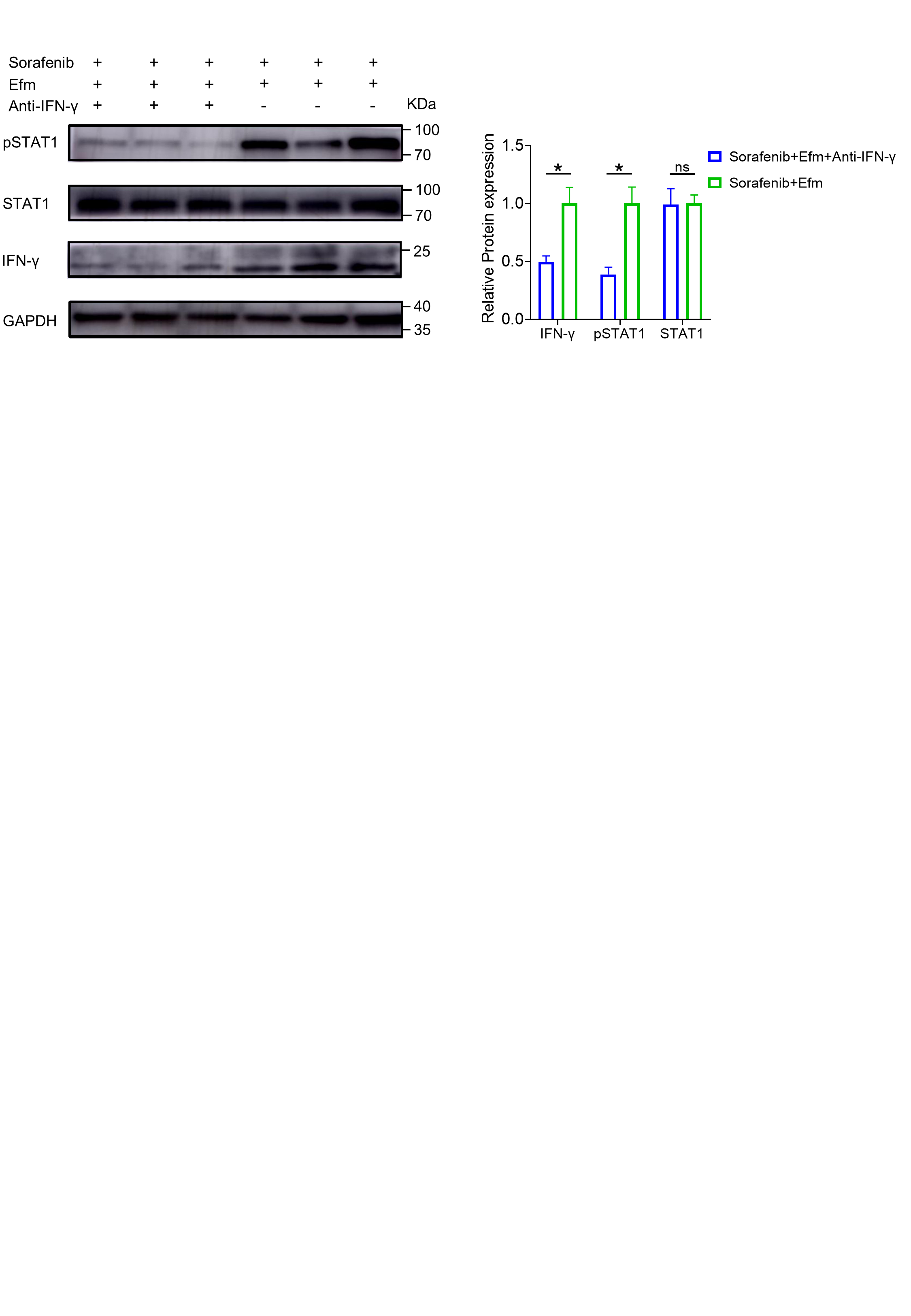
Figure S4**

**Fig S4**. The relative protein expression of IFN-γ, STAT1 and pSTAT1 in tumor tissues was evaluated by Western blotting, with and without the use of anti-mouse IFN-γ antibody. Error bars represent the SEM. ns P>0.05, * P<0.05.
